# Humanitarian crises and child-marriage: Historical recurrent interrelated events

**DOI:** 10.7189/jogh.11.03112

**Published:** 2021-10-30

**Authors:** Saverio Bellizzi, Liliana Lorettu, Gabriele Farina, Luciano Bubbico, Salvatore Ferlito, Andrea Cegolon, Giuseppe Pichierri, Luca Cegolon

**Affiliations:** 1 Medical Epidemiologist, Independent Consultant, Geneva, Switzerland; 2 University of Sassari, Sassari, Italy; 3 Department of Sensorineural Disabilities, INAPP/Italian Institute of Social Medicine, Rome, Italy; 4 University of Catania School of Medicine, Department of Surgical Medical Sciences and Advanced Technologies, Catania, Italy; 5 Department of Political, Social and International Relationships, University of Macerata, Macerata, Italy; 6 Microbiology Department, Kingston Hospital NHS Foundation Trust, Kingston upon Thames,; London, UK; 7 University of Trieste, Department of Medical, Surgical & Health Sciences, Trieste, Italy

Child marriage occurs when at least one of the partners is under 18 years of age and is a form of violence against women and girls (VaWG), as described in the Convention on the Rights of Children [1]. While nearly all of the world’s countries have legal minimum age requirements to protect children from child marriages, at least 117 of these countries still allow children to marry, according to a 2016 survey by the Pew Research Center [2].

Child marriage is associated with events like unintended births and pregnancy termination (childbirth is the leading cause of death among girls aged 15-19 years) as well as school dropouts, thus affecting the growth and well-being particularly of young girls [3]. Due to the aforementioned reasons, governments around the world have committed to eliminate child marriage by 2030, by including ending child marriage a target under the United Nations Sustainable Development Goals 5.3 and 16.2.

Progress to achieve this objective has however been uneven so far: while South Asia has led the way in reducing child marriage over the last decade, the overall global burden has shifted to Sub-Saharan Africa, where one in three recently married girls reside, compared to one in five a decade ago, and where rates of progress need to be scaled up dramatically to offset population growth [4]. Slightly less than 80 million child marriages have been prevented over the past 25 years through policies and programming [5]. While a steep acceleration in progress was achieved during 2000-2010 with a 41.0% decrease in child marriages, such a progress has flattened in the last ten years [6].

A new analysis from World Vision revealed that between March and December 2020 child marriages more than doubled compared to 2019 in several communities in Afghanistan, Bangladesh, Senegal and Uganda [7]. Before the pandemic, India, which accounts for one in three child marriages globally, had become a world leader in working to reduce child marriage, through education and awareness; however, child marriages reportedly increased by more than 33% between June and October 2020, compared to the same period in 2019 [8]; the Indian Minister for Women and Child Development Smriti Irani tweeted that Childline helped prevent 898 child marriages during the 2020 lockdown [7]. Similarly, child advocates are particularly concerned in Nepal where teen pregnancies have nearly tripled [9,10]. The situation is particularly alarming in Bangladesh where an assessment on March 11 2021 by the Manusher Jonno Foundation (MJF) in association with the UN Population Fund, UNICEF and Plan International Bangladesh reported at least 13 886 girls in 21 districts were victims of child marriages between April and October of 2020; 48% out of the total were between 13 and 15 years old [11]. The negative effect of the SARS-CoV-2 pandemic on child marriage is not surprising (2.5 million girls at risk around the world in the next 5 years and as many as one million more girls at risk of becoming pregnant this year alone) [12].

Experience from past historical situations is compelling: numerous complex factors that may drive child marriage in stable environments are greatly exacerbated in emergency settings, as family and community structures break down during crisis and displacement with loss of household income and higher risk of violence in the household. There is clear literature on the effect of disasters on poor families, which in times of crisis end up marrying their underage children as a way to pursue other sources of income or reduce the current household burden [13]. Despite limited data, girls affected by humanitarian crises such as conflicts, droughts, earthquakes and outbreaks, face the greatest risks of child marriage. In fact, nine of the ten countries with the highest rates of child marriage are considered fragile states, including Afghanistan where 57 percent of girls were married before the age of 19 in pre-COVID-19 times [14]. In 2018 an earthquake and tsunami struck Palu, a city on the island of Sulawesi in Indonesia; the next year cases of child marriage rose from 14 to 48 in just one village, according to libu Perempuan (lp), a women’s-rights group [12]. In Nepal, child marriages reportedly rose as destruction of houses and property and interruption of livelihoods from the 2015 earthquake resulted in additional financial strain [15,16].

Evidence on the increasing prevalence of child marriage is particularly striking in war zones such as South Sudan and Yemen, where a 20.0% rise has been reported as a result of conflicts; in such contexts, girls and women are at particular risk of sexual violence and trafficking and child marriage can be used as a cover for this [17]. On the other hand, young girls may engage in informal unions with older men in the hope of reuniting with members of their families that have already migrated to other countries [17]. The same issue does not spare refugee communities in their host countries: in Jordan, 12.0% of registered marriages involved a girl under the age of 18 in 2011; prevalence rose to 18.0% in 2012, 25.0% in 2013 and 32.0% in early 2014 [18]. Similarly, 41.0% of young, displaced women in 2018 in Lebanon were married before 18 [19].

**Figure:**
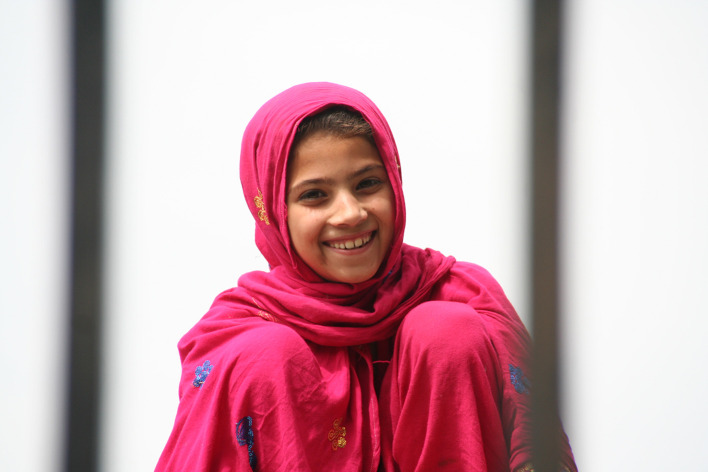
Photo: Young girl in Pakistan (from Saverio Bellizzi’s own collection, used with permission).

Disease outbreaks represent other typical situations featuring increasing patterns of early marriages. Lockdowns that are implemented to decrease transmission of SARS-CoV-2 are associated with increases in violence against women and girls [20], in addition, many girls will never return to school due to increasing pressure to work, risk of child marriage, bans on pregnant girls attending school and lost contact with education.

The current increase which marks a significant surge in child marriage rates (61 million child marriages by 2025) is set to reverse 25 years of progress, which saw child marriage rates decline [12]. Reopening schools, implementing effective laws and policies, providing comprehensive social protection measures for families, ensuring access to sexual and reproductive health services and social services remain critical to significantly reduce a girl’s risk of having her childhood stolen through child marriage [21]. Also, it is fundamentally important to change the view of several societies that marrying children solves adolescent promiscuity, prevents pregnancy, and addresses the issue of non-marital pregnancy [9]. Governments and International Partners should address child marriage in assessments and programming. Early marriage must be integrated within the child protection and gender-based violence (GBV) areas of responsibility, ensuring a comprehensive and multi-sectoral response for all young women. To this end, participation of community-based organization working on gender equality and rights of children is critical in order to identify and respond to the needs of girls. Specifically, emergency preparedness and response plans, including those for COVID-19, must embed the view and participation of women and young girls together with the inclusion of essential services to prevent and manage VaWG. On the other hand, financial assistance must be a core component of plans and strategies to build back better. It is noteworthy that only 0.12% of funding in humanitarian contexts goes to issues related to gender-based violence, including child marriage. Also, only 0.58% of the budget for the global plan to respond to COVID-19 in areas affected by humanitarian crises is allocated to gender-based violence [6]. Finally, the availability of frequently updated and disaggregated data on gender-based violence and child marriage is critical to ensure adequate preparedness, mitigation, and localized response.
